# Contribution of *Candida* biomarkers and DNA detection for the diagnosis of invasive candidiasis in ICU patients with severe abdominal conditions

**DOI:** 10.1186/s13054-016-1324-3

**Published:** 2016-05-16

**Authors:** Cristóbal León, Sergio Ruiz-Santana, Pedro Saavedra, Carmen Castro, Ana Loza, Ismail Zakariya, Alejandro Úbeda, Manuel Parra, Desirée Macías, José Ignacio Tomás, Antonio Rezusta, Alejandro Rodríguez, Frederic Gómez, Estrella Martín-Mazuelos, The Cava Trem Study Group

**Affiliations:** Intensive Care Unit, Hospital Universitario de Valme, Universidad de Sevilla, Avenida Bellavista s/n, 41014 Sevilla, Spain; Intensive Care Unit, Hospital Universitario Dr. Negrín, Universidad de Las Palmas de Gran Canaria, Las Palmas de Gran Canaria, Spain; Mathematics Department, Universidad de las Palmas de Gran Canaria, Las Palmas de Gran Canaria, Spain; Clinical Unit of Microbiology and Infectious Diseases, Hospital Universitario de Valme, Universidad de Sevilla, Sevilla, Spain; Intensive Care Unit, Hospital Punta de Europa, Algeciras, Cádiz Spain; Intensive Care Unit, Hospital Universitario Miguel Servet, Zaragoza, Spain; Service of Microbiology, Hospital Universitario Miguel Servet, Zaragoza, Spain; Critical Care Department, Hospital Universitari Joan XXIII, Tarragona, Spain; Service of Microbiology, Hospital Universitari Joan XXIII, Tarragona, Spain

**Keywords:** Invasive candidiasis, *Candida* spp. colonization, (1 → 3)-ß-D-glucan, *Candida albicans* germ tube antibody, Mannan antigen, Anti-mannan antibody, Candida PCR, Intra-abdominal candidiasis, Candidemia

## Abstract

**Background:**

To assess the performance of *Candida albicans* germ tube antibody (CAGTA), (1 → 3)-ß-D-glucan (BDG), mannan antigen (mannan-Ag), anti-mannan antibodies (mannan-Ab), and *Candida* DNA for diagnosing invasive candidiasis (IC) in ICU patients with severe abdominal conditions (SAC).

**Methods:**

A prospective study of 233 non-neutropenic patients with SAC on ICU admission and expected stay ≥ 7 days. CAGTA (cutoff positivity ≥ 1/160), BDG (≥80, 100 and 200 pg/mL), mannan-Ag (≥60 pg/mL), mannan-Ab (≥10 UA/mL) were measured twice a week, and *Candida* DNA only in patients treated with systemic antifungals. IC diagnosis required positivities of two biomarkers in a single sample or positivities of any biomarker in two consecutive samples. Patients were classified as neither colonized nor infected (*n* = 48), *Candida* spp. colonization (*n* = 154) (low-grade, *n* = 130; high-grade, *n* = 24), and IC (*n* = 31) (intra-abdominal candidiasis, *n* = 20; candidemia, *n* = 11).

**Results:**

The combination of CAGTA and BDG positivities in a single sample or at least one of the two biomarkers positive in two consecutive samples showed 90.3 % (95 % CI 74.2–98.0) sensitivity, 42.1 % (95 % CI 35.2–98.8) specificity, and 96.6 % (95 % CI 90.5–98.8) negative predictive value. BDG positivities in two consecutive samples had 76.7 % (95 % CI 57.7–90.1) sensitivity and 57.2 % (95 % CI 49.9–64.3) specificity. Mannan-Ag, mannan-Ab, and *Candida* DNA individually or combined showed a low discriminating capacity.

**Conclusions:**

Positive *Candida albicans* germ tube antibody and (1 → 3)-ß-D-glucan in a single blood sample or (1 → 3)-ß-D-glucan positivity in two consecutive blood samples allowed discriminating invasive candidiasis from *Candida* spp. colonization in critically ill patients with severe abdominal conditions. These findings may be helpful to tailor empirical antifungal therapy in this patient population.

## Background

Accurate and timely diagnosis of invasive candidiasis (IC) from the patient’s perspective and to optimize antifungal therapy in the intensive care unit (ICU) setting remains a topic of great interest [1–6]. The use of single or combined biomarkers, such as (1 → 3)-ß-D-glucan (BDG), *Candida albicans* germ tube antibody (CAGTA), mannan antigen (mannan-Ag), anti-mannan antibodies (mannan-Ab), and polymerase chain reaction (PCR) detection of *Candida* DNA has received increasing attention [7–10], but the appropriate incorporation into clinical practice remains controversial. We investigated the performance of these five tests, alone and in combination, for discriminating IC in critically ill patients with severe abdominal conditions (SAC).

## Methods

### Design and study population

Between November 1, 2012 and February 28, 2014, all consecutive adult non-neutropenic patients with SAC on ICU admission and an expected stay of ≥ 7 days were included in a prospective, cohort, observational, and multicenter study. The study protocol was approved by the Ethics Committee of Hospital Universitario de Valme (Sevilla, Spain) and the Spanish Agency for Medicines and Health Care Products (AEMPS). The codes and dates of approval of the study protocol were CEIC-A1, ref. 350/12 (May 29, 2012) for the Ethics Committee of Hospital de Valme, and September 14, 2012 for AEMPS. Informed consent was obtained from the patients or their legal representatives.

A severe abdominal condition (SAC) was defined as the process that caused gastrointestinal dysfunction or failure in the context of a medical abdominal disease (e.g., severe acute pancreatitis) or an abdominal surgical condition requiring elective or urgent procedures, with related complications (e.g., gastrointestinal perforation, hepatobiliary and pancreatic disorders, peritonitis, intra-abdominal abscess, anastomotic leak, etc.) and prolonged postoperative stay after complicated abdominal surgery. Therefore, the definition of SAC included medical and surgical patients. Data for each patient was recorded using an electronic case report form.

Besides neutropenia defined as total leukocyte count < 1000/mm3, other exclusion criteria on ICU admission were as follows: age below 18 years, human immunodeficiency virus (HIV) infection, active malignancy, current immunosuppression or immunosuppressive therapy, treatment with immunomodulating agents (monoclonal antibodies) in the previous 3 months, use of a dose of ≥ 20 mg/day of prednisone or its equivalent within 1 month prior to ICU admission, spontaneous bacterial peritonitis in liver cirrhosis or advanced chronic liver disease, solid organ or bone marrow transplant recipients, chronic inflammatory bowel disease (ulcerative colitis, Crohn’s disease), life expectancy less than 1 week, Acute Physiology and Chronic Health Evaluation (APACHE II) score > 35 on ICU admission, documented *Candida* spp. infection during the week prior to ICU admission, treatment with antifungal agents before ICU admission or before inclusion in the study, limitation of the therapeutic effort, refusal to sign the informed consent, and inadequate data collection (incompleteness of the protocol specifications).

### Screening, microbiological cultures, and *Candida* score

Surveillance cultures for the screening *Candida* spp. were performed twice a week from the fourth day of ICU admission. Surveillance samples were obtained from feces or rectal swabs, urine, tracheal aspirates (or protected specimen brush or bronchoalveolar lavage), oropharyngeal swabs (in patients without mechanical ventilation), peripheral blood, vascular lines, wound/drainage exudates, or infected foci at the discretion of the attending physician. Samples were seeded directly into *Candida* CHROMagar™ Chromogen culture medium (Hardy Diagnostics, Santa Maria, CA, USA). All catheter tips removed were cultured in blood agar and Sabouraud agar by the Maki roll plate technique. Blood cultures were processed using the automated BACTEC™ system (Becton Dickinson Diagnostic Instrument System, Paramus, NJ, USA) or other standardized methods. Results were considered positive in the presence of *Candida* growth in the culture medium. Identification at the species level was required. *Candida* score [11, 12] was calculated coinciding with collection of samples and once culture data were available.

### Serological biomarkers

Blood samples (15 mL) were collected in three tubes without anticoagulant, centrifuged at 1800 rpm for 10 min, separated into aliquots, and stored at –80 °C until analysis. None underwent more than one freeze–thaw cycle, and serum and reagents were tempered and homogenized before processing.

The BDG assay (Fungitell™, Associates of Cape Cod, Inc., East Falmouth, MA, USA) was performed according to the manufacturer’s recommendations: under laminar flow hood, duplicated and saving the mean value of both measurements and repeating the assay when between both BDG values there existed at least a 20 % difference. Also, when the BDG values were greater than 500 pg/mL, samples were diluted and retested. The cutoff value was ≥ 80 pg/mL. CAGTA detection was performed by an immunofluorescence test (Vircell kit assay, Granada, Spain) according to the manufacturer’s instructions. The cutoff value for positive CAGTA was ≥ 1/160. For mannan-Ag and mannan-Ab, Platelia *Candida* Ag Plus and Platelia *Candida* Ab Plus were used in the automated EVOLISTM Twin Plus device (Bio-Rad, Marnes-la-Coquette, France). The cutoff values for positive mannan-Ag and mannan-Ab were ≥ 60 pg/mL and ≥ 10 AU/mL, respectively. None of the tests were performed in real time and, therefore, were not available to clinician’s decision making.

### Multiplex quantitative real-time PCR (MRT-PCR)

The MRT-PCR assay [13] was performed to detect the six most frequent species of the genus *Candida* in invasive candidiasis (IC). The technique detected *C. albicans, C. parapsilosis, C. tropicalis, C. glabrata, C. krusei*, and *C. guilliermondii* using specific molecular beacon probes labeled with different fluorescent dyes: FAM, HEX, ROX and CYAN 500. Primers and probes were designed on the basis of the nucleotide sequences of the ITS ribosomal DNA region from strains belonging to the collection of the Spanish National Center of Microbiology. The probes targeted the ITS1 or ITS2 regions of ribosomal DNA. These regions were chosen as targets because of the possibility of designing a suitable probe for each case. Beacon Designer 5.0 software (Premier Biosoft, Palo Alto, CA, USA) was used to design primers and probes. The assay consisted of two multiplex PCRs: reaction one (*C. albicans, C. parapsilosis, and C. tropicalis*), which was performed using the LightCycler Probes Master Kit (Roche Diagnostics, Madrid, Spain); and reaction two (*C. glabrata, C. krusei, and C. guilliermondii*), which was performed using the 2x Sensimix Probe Kit (Quantace, Ecogen, Madrid, Spain). Both PCRs were performed simultaneously in the LightCycler 480 System (Roche Diagnostics, Mannheim, Germany). DNA from blood and sera was extracted using the QIAamp DNA Mini Kit (Qiagen Izasa, Madrid, Spain). Elution was performed with 50 mL of buffer; the PCR was performed with 2 mL of DNA extracted from each sample. All samples were performed in duplicate and quantification standards were run in conjunction with each set of samples and negative controls.

### Study protocol and collection of data

The following variables were recorded: age, sex, reason for ICU admission, APACHE II score, Sepsis-related Organ Failure Assessment (SOFA) score on ICU admission, comorbidities, and risk factors associated with *Candida* spp. colonization or infection. According to diagnosis at the time of ICU admission, patients were classified as medical or surgical. Surgical patients were those for whom the reason of ICU admission was the postoperative control of an elective or urgent surgical procedure. All surgical procedures and the number of operations performed in each patient were recorded. Medical patients undergoing major surgery during ICU admission were considered surgical patients.

Underlying diseases included diabetes mellitus treated with oral hypoglycemic agents and insulin-dependent diabetes, chronic obstructive pulmonary disease (COPD) (airflow limitation defined as forced expiratory volume during the first second (FEV_1_)/forced vital capacity (FVC) < 88 % predicted in men and < 89 % predicted in women, or a postbronchodilator ratio of FEV_1_/FVC < 0.7), chronic liver disease (confirmed by liver biopsy or the presence of signs of portal hypertension, such as esophageal varices or ascites), chronic renal failure (in patients requiring hemodialysis or peritoneal dialysis), severe heart failure (defined as grades III and IV according to the New York Heart Association [NYHA] classification, alcoholism (defined as ethanol ingestion > 80 g/day), and HIV infection with adequate clinical control.

Risk factors for the development of *Candida* spp. colonization or infection included the following: central venous catheters, arterial catheters, total parenteral nutrition, mechanical ventilation, continuous renal replacement therapy (hemofiltration), treatment with corticosteroids (defined as intravenous administration of steroids for 5 days or more), and broad-spectrum antimicrobial treatment.

Once the patient was included in the study, the following data were recorded twice a week from the fourth day of ICU stay and for 3 weeks: surveillance cultures, Acute Physiology and Chronic Health Evaluation (APACHE II) score, Sepsis-related Organ Failure Assessment (SOFA) score, clinical situation assessment (presence or absence of sepsis, severe sepsis, or septic shock), and *Candida* score. Blood samples for the measurement of BDG, CAGTA, mannan-Ag, and mannan-Ab were drawn at the same time periods. In patients treated with empirical or directed systemic antifungal treatment (SAT), clinical and microbiological study controls were performed on days 0, +3, +7, and +14 from the beginning of SAT. In addition, blood cultures and samples for PCR detection of *Candida* DNA were also obtained. Patients were followed until ICU and/or hospital discharge, or death. The indication of SAT was decided by the primary clinician. Details of SAT including starting date, drug(s), doses and duration of treatment were registered.

### Definitions

Catheter-related candidemia was diagnosed in those patients who had an intravascular device and one or more positive cultures of blood samples obtained from the peripheral vein, clinical manifestations of infection (e.g., fever, chills, and/or hypertension), and no apparent source for bloodstream infection (with the exception of the catheter), as well as a positive catheter culture, either semiquantitative culture (≥15 colony-forming units [cfu] per catheter segment) or quantitative (≥1000 cfu per catheter segment), whereby the same organism (species and susceptibility) were isolated from a catheter segment and a peripheral blood sample. Candiduria was defined in the presence of at least 10^4^ cfu/mL of the same *Candida* spp.

When BDG, CAGTA, mannan-Ag, and mannan-Ab were assessed independently, positivity for IC required values of any biomarker at or above the cutoff level in two consecutive samples. *Candida* PCR (C-PCR) was considered positive when *Candida* DNA was detected at least in a single sample. When the combination of two biomarkers was assessed, positivity for IC required values of both biomarkers at or above the cutoff level in a single sample, or values at or above the cutoff level for at least one of these biomarkers in two consecutive samples.

*Candida* colonization was considered unifocal when *Candida* spp. was isolated from one site and multifocal when *Candida* spp. was simultaneously isolated from various noncontiguous sites, even if two different *Candida* spp. were isolated. Low-grade *Candida* colonization was defined when *Candida* spp. was isolated in one or more foci, in one or two consecutive surveillance controls. High-grade *Candida* spp. colonization was defined as colonization of at least three body sites on two or more consecutive screening days [14]. Invasive candidiasis or proven *Candida* infection was defined as (i) primary candidemia (presence of *Candida* spp. in one or more blood cultures obtained from peripheral veins), and (ii) intra-abdominal candidiasis (IAC) (on the basis of macroscopic findings and direct examination or positive culture for *Candida* spp. of the peritoneal fluid collected during operation or within 24 h from external drainage) [15].

Patients were classified into the groups of neither colonized nor infected, low-grade *Candida* spp. colonization, high-grade *Candida* spp. colonization, and IC. In the presence of candidemia, catheters were removed, and fundoscopy was performed.

**Fig. 1:**
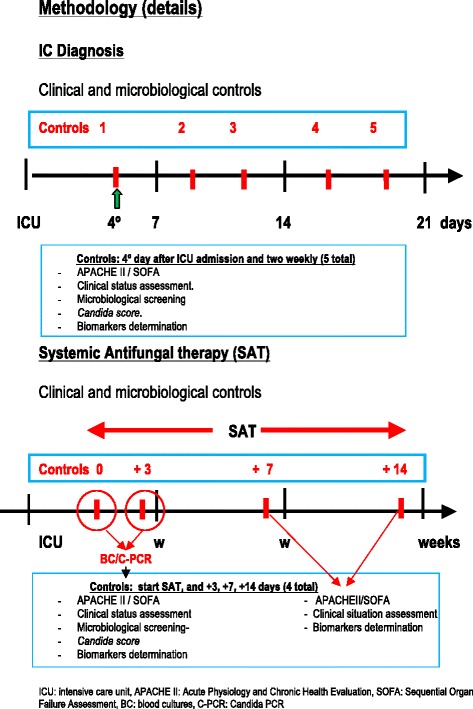
Details of the study methodology

Clinical, microbiological, and serological controls performed during the diagnostic phase are shown in Fig. 1. When the patient received systemic antifungal therapy (SAT) (treatment phase), controls were performed at different time schedules and detection of *Candida* DNA by PCR assay was included in the two first determinations.

### Statistical analysis

Categorical variables are expressed as frequencies and percentages, and continuous variables as mean and standard deviation (SD) when data followed a normal distribution, or as median and interquartile range (25th–75th percentile) when distribution departed from normality. The percentages were compared using the chi-square (χ^2^) test, the means by the F test, and the medians by the Kruskal-Wallis test. Statistical significance was set at *P* < 0.05. The performance of the BDG assay was analyzed with ≥ 100 and ≥ 200 pg/mL cutoffs besides the cutoff of ≥ 80 pg/mL. Sensitivity, specificity, and predictive values for the ability of each biomarker to discriminate between the IC and the remaining groups were calculated. Data were analyzed using the R package, version 3.1.0 (R Development Core Team, 2014) [16].

## Results

### Study population and salient findings

**Fig. 2:**
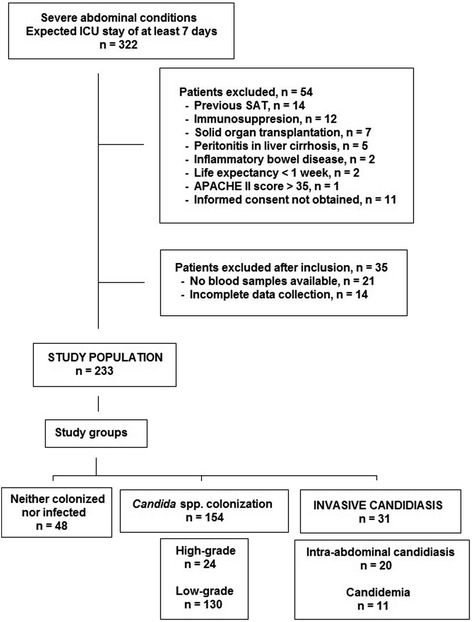
Flow-chart of the study population

The flow chart of the study population is shown in Fig. 2. Of 322 eligible patients, 89 (27.6 %) were excluded for different reasons (Fig. 2). Data of 233 patients (67 % men, mean age 66.7 years) were analyzed. In relation to SAC, 211 patients underwent 426 operations (66 % on an emergency basis; 50 % related to the colon, biliary tract, and pancreas; 37 % reoperations) and 22 had acute pancreatitis. Details of surgical procedures are shown in Table 1.

**Table 1:** Details of surgical procedures

Operation	Surgical procedures			Total
	1st	2nd	3rd	
Surgery of the esophagus				
Esophagectomy with/without gastrectomy	13	4	2	19
Surgery of the stomach				
Gastric bypass (gastroduodenostomy, gastrojejunal, Roux-en-Y)	7	7	1	15
Radical subtotal gastrectomy	4	2		6
Total gastrectomy	4	1	1	6
Partial gastrectomy plus vagus nerve transection	2			2
Gastrorraphy		1		1
Gastrostomy	1			1
Surgery of the gallbladder, biliary tract, and liver				
Cholecystectomy	26	1		27
Biliary tract surgery (cholecystostomy, bypasses, sphincteroplasty, etc.)	11	4	5	20
Segmental hepatectomy	6			6
Resection hydatid cyst	3			3
Open drainage of liver abscess	1		1	2
Resection hepatic tumor	1			1
Multiple or simple hepatorrhaphy	1			1
Surgery of the pancreas				
Duodenocephalic pancreatectomy	11	2		13
Drainage pancreatic abscess	9	1		10
Distal, subtotal pancreatectomy or resection of pancreatic lesions	5	1	1	7
Surgery of the small intestine and colon				
Intestinal resection: duodenectomy, enterocolectomy, enterectomy, jejunectomy	34	10	2	46
Subtotal colectomty (includes hemicolectomy and sigmoidectomy)	37	5	1	43
Colostomy or ileostomy (includes sigmoidostomy) duodenostomy	16	19	4	39
Intestinal anastomoses without resection (small intestine, small-large intestine, large intestine)	15	4	2	21
Total colectomy	7			7
Appendectomy	3			3
Closure of stoma	1	1	1	3
Drainage of diverticular abscess	2			2
Reduction of intestinal volvolus. Intestinal intussusception	2			2
Meckel’s diverticulum resection	1			1
Retroperitoneum				
Drainage of intraperitoneal abscess (including epiploic, iliac fossa, perisplenic and perigastric)	5	12		17
Enterotomy: foreign body	2			2
Drainage retroperitoneal abscess	1	1		2
Other				
Exploratory laparotomy	29	32	18	79
Drainage of abdominal wall abscess	2	2	2	6
Hernia repair with/without mesh	2	2		4
Splenectomy. Splenorrhaphy		1	1	2
Drainage subphrenic or subdiaphragmatic abscess	1		1	2
Resection lesion in the mesenterium or omentum	1			1
Aortobifemoral bypass		1		1
Endovascular repair of abdominal aortic aneurysm	1			1
Placement Bogota bag			1	1
Ileofemoral thromboendarterectomy	1			1
			Total	426

Thirty-one patients had IC (IAC 20; candidemia 11), 154 *Candida* spp. colonization (low-grade 130; high-grade 24), and 48 were neither colonized nor infected. The groups of IAC and high-grade colonization showed significant differences as compared to the remaining groups in *Candida* score, length of ICU stay, and number of surgical procedures; also, antifungal treatment was significantly more frequent among patients with IC (Table 2).

**Table 2:** Characteristics of the study population according to colonization and infection status

	Diagnosis						*P* value
	Total	NCI	LGCC	HGCC	IAC	Candidemia	
	(*n* = 233)	(*n* = 48)	(*n* = 130)	(*n* = 24)	(*n* = 20)	(*n* = 11)	
Age, years, mean (± SD)	66.7 ± 13.2	66.9 ± 11.9	65.8 ± 13.9	70.7 ± 9.2	67.1 ± 15.7	67.5 ± 12.2	.597
Male/female, %	67.0/33.0	72.9/27.1	70.0/30.0	50.0/50.0	60.0/40.0	54.5/45.5	.228
ICU admission							
APACHE II	18.4 ± 6.3	18.1 ± 6.2	18.5 ± 6.1	18.2 ± 7.1	19.6 ± 7.0	18.3 ± 6.5	.925
SOFA^a^	7 (5 ; 9)	6.5 (5 ; 8)	7 (5 ; 10)	7.5 (4.5; 9.5)	6.5 (4 ; 10)	7.5 (5 ; 9)	.583
Maximum score during ICU stay							
APACHE II	18.7 ± 6.3	17.7 ± 6.6	18.3 ± 6.1	21.4 ± 7.7	19.4 ± 5.0	19.5 ± 4.7	.162
SOFA^a^	7 (5–10)	7 (4–9)	7 (5–10)	7 (5–10)	7 (5–12)	9 (5.5–10)	.702
*Candida* score^*a*^	4 (3–4)	3 (2–4)	4 (3–4)	4 (3–5)	4.5 (4–5)	3 (3–4)	< .001
ICU length of stay, days^a^	15 (10–25)	12 (8–19)	14 (9–25)	21 (17–28)	14 (11–19)	16 (8–38)	.017
Hospital length of stay, days^a^	37 (21–57)	34 (22–45)	33 (19–59)	48 (33–68)	37 (26–58)	40 (32–59)	.097
Patient type, no. (%)							.180
Medical	22 (9.4)	5 (10.4)	12 (9.2)	2 (8.3)	0	3 (27.3)	
Surgical^*^	211 (90.6)	43 (89.6)	118 (90.8)	22 (91.7)	20 (100)	8 (72.7)	
Pancreatitis not intervened	22 (9.4)	5 (10.4)	12 (9.2)	2 (8.3)	0	3 (27.3)	.232
Site of abdominal surgery, no. (%)							.701
Esophagus	13 (6.2)	4 (9.3)	5 (4.2)	2 (9.1)	2 (10.0)	0	
Stomach/duodenum	21 (10.0)	1 (2.3)	14 (11.9)	3 (13.6)	3 (15.0)	0	
Biliary tract/pancreas	58 (27.5)	9 (20.9)	38 (32.2)	5 (22.7)	3 (15.0)	3 (37.5)	
Small intestine	45 (21.3)	13 (30.2)	21 (17.8)	4 (18.2)	5 (25.0)	2 (25.0)	
Colon	59 (28.0)	12 (27.9)	32 (27.1)	6 (27.3)	6 (30.0)	3 (37.5)	
Others	15 (7.1)	4 (9.3)	8 (6.8)	2 (9.1)	1 (5.0)	0	
Surgery type^**^, no. (%)^†^							.556
Emergency	135 (64.0)	24 (55.8)	78 (66.1)	13 (59.1)	15 (75.0)	5 (62.2)	
Elective	76 (36.0)	19 (44.2)	40 (33.9)	9 (40.9)	5 (25.0)	3 (37.5)	
Surgical procedure^**^, no. (%)							.023
One	113 (53.6)	23 (53.5)	70 (59.3)	11 (50.0)	4 (20.0)	5 (62.5)	
≥ Two	98 (46.4)	20 (46.5)	48 (40.7)	11 (50.0)	16 (80.0)	3 (37.5)	
Underlying illnesses, no. (%)							
Solid tumor	84 (36.1)	19 (39.6)	43 (33.1)	10 (41.7)	8 (40.0)	4 (36.4)	.853
Diabetes mellitus	67 (28.8)	13 (27.1)	35 (26.9)	8 (33.3)	7 (35.0)	4 (36.4)	.830
COPD	37 (15.9)	13 (27.1)	17 (13.1)	3 (12.5)	4 (20.0)	0	.112
Chronic renal failure	26 (11.2)	3 (6.2)	14 (10.8)	5 (20.8)	2 (10.0)	2 (18.2)	.337
Heart failure	16 (6.9)	3 (6.2)	6 (4.6)	5 (20.8)	0	2 (18.2)	.023
Chronic liver failure	9 (3.9)	4 (8.3)	3 (2.3)	2 (8.3)	0	0	.201
Oncohematologic process	3 (1.3)	0	2 (1.5)	1 (4.2)	0	0	.635
Antifungal treatment, no. (%)	119 (51.1)	21 (43.8)	58 (44.6)	11 (45.8)	18 (90.0)	11 (100)	< .001
ICU mortality, no. (%)	50 (21.5)	8 (16.7)	30 (23.1)	7 (29.7)	4 (20.0)	1 (9.1)	.605
Overall mortality, no. (%)	68 (29.2)	10 (20.8)	41 (31.5)	8 (33.3)	8 (40.0)	1 (9.1)	.277

Values are expressed as frequencies and percentages; mean ± standard deviation (SD); ^a^median (25th-75th percentiles); *P* value: statistical significance

*NCI* neither colonized nor infected, *LGCC* low-grade *Candida* spp. colonization, *HGCC* high-grade *Candida* spp. colonization, *IAC* intra-abdominal candidiasis, *ICU* intensive care unit, *APACHE II* Acute Physiology and Chronic Health Evaluation, *SOFA* Sequential Organ Failure Assessment, *COPD* chronic obstructive pulmonary disease

^*^Abdominal surgery; ^**^211 patients, ^†^first surgical procedure

Data on risk factors, *Candida* colonization, CI, and SAT are shown in Table 3. Twenty-three (74.1 %) of the 31 patients with IC had sepsis or septic shock, with a median time between ICU admission and diagnosis of infection of 7 days. The most common causative pathogens were *C. albicans* (51.6 %) and *C. glabrata* (22.6 %). Of the 31 patients with IC, 29 (93.5 %) received SAT (ICA 18, candidemia 11) and 2 died before indication of antifungal therapy. The median time between ICU admission and beginning of SAT was similar for patients with candidemia (6.5 days) and patients with IAC (7 days). Empirical SAT was administered in 90 patients (75.6 %), with severe sepsis or septic shock in 65, and a median time between ICU admission and starting treatment of 7 days. Patients treated with SAT for suspected or documented *Candida* spp. infection showed similar characteristics, except for a higher *Candida* score in patients with IC (median [IQR] 4 [3, 4] vs. 4 [3–5], *P* = 0.031).

**Table 3:** Risk factors, *Candida* colonization (low and high grade) and infection, and antifungal therapy characteristics of patients

Included patients	*n* = 185^*^
Risk factors, no. (%)	
Broad-spectrum antibiotics	184 (99.5)
Urinary catheter	183 (98.9)
Central venous catheter	183 (98.9)
Arterial catheter	165 (89.2)
Mechanical ventilation	156 (84.3)
Parenteral nutrition	155 (83.8)
Corticosteroids	73 (39.5)
Renal replacement therapy	54 (29.2)
Candida colonization (*n* = 154)	
Low-grade *Candida* spp. colonization, no. (%)	130 (84.4)
High-grade *Candida* spp. colonization, no. (%)	24 (15.6)
Candida score ≥ 3; first week/during study	96/142 (51.9)
Candida infection (*n* = 31)	
Severity of *Candida* infection at diagnosis	
No sepsis/sepsis	3/5
Severe sepsis/septic shock	9/14
Causative *Candida spp*	
*C. albicans*	16 (51.6)
*C. glabrata*	7 (22.6)
*C. parapsilosis*	3 (9.7)
*C. tropicalis*	1 3.2)
*C. krusei*	1 (3.2)
*C. dubliniensis*	1 (3.2)
*C. famata*	1 (3.2)
*C. albicans + C. glabrata*	1 (3.2)
Time between hospital admission to infection, days^a^	16 (6.5–19.5)
Time between ICU admission to infection, days^a^	7 (2–13)
Hospital/ICU mortality, no. (%)	9 (29.0/5 (16.1)
Systemic antifungal therapy (SAT) (*n* = 119)^**^	
Empirical therapy for suspected IC (*n* = 90)^***^	
Multifocal colonization, no. (%)	50 (55.6)
Clinical situation, no. (%)	
No sepsis/sepsis	8 (8.9)/17 (18.9)
Severe sepsis/septic shock	29 (32.2)/36 (40.0)
APACHE II	17.8 ± 6.2
SOFA^a^	6 (5–9)
Candida score	4 (3–4)
Time between of hospital/and beginning of SAT, days^a^	11 (7–19)
Time between ICU admission and beginning of SAT, days^a^	7 (1–11)
Hospital/ICU mortality, no. (%)	29 (32.2)/23 (25.6)
Therapy for documented infection (*n* = 29)^***^	
Clinical situation	
No sepsis/sepsis	3 (10.3)/4 (13.8)
Severe sepsis/septic shock	8 (27.6)/14 (48.3)
APACHE II	18.8 ± 6.8
SOFA^a^	6.5 (4–10)
Candida score	4 (3–5)
Time between of hospital/ICU admission and beginning of SAT, days^a^	
Candidemia (11)	15 (4.5 ; 22)/6.5 (1 ; 16)
Intra-abdominal candidiasis (18)	12.5 (8 ; 17)/7 (3 ; 12)
Antifungal agents (>1 agent/patient)	
Days of therapy (first SAT)^a^	15 (8–21)

Values are expressed as frequencies and percentages; mean ± standard deviation (SD); ^a^median (interquartile range, 25th–75th percentile)

*ICU* intensive care unit, *SAT* systemic antifungal therapy, *IC* invasive candidiasis, *APACHE II* Acute Physiology and Chronic Health Evaluation, *SOFA* Sequential Organ Failure Assessment

^*^Patients with *Candida* colonization (low and high grade) (*n* = 154) plus invasive candidiasis (*n* = 31), ^**^patients with *Candida* colonization (*n* = 90) and IC (*n* = 29); ^***^starting antifungal therapy

### Diagnostic value of BDG (cutoffs 80, 100 and 200 pg/mL), CAGTA, mannan-Ag, mannan-Ab, and C-PCR alone

BDG, CAGTA, mannan-Ag, and mannan-Ab were measured in 860 samples (3.6 per patient), and C-PCR in 213, with positive results in 453, 306, 287, 150, and 110 samples, respectively. As shown in Tables 4 and 5, the percentages of patients with positive BDG, CAGTA, and mannan-Ag were significantly higher in the groups of candidemia, IAC, and high-grade *Candida* spp. colonization than in the remaining groups. When the BDG assay was used with the different cutoffs, the number of patients with positive results remained without changes in the group with candidemia but decreased in the groups of high-grade *Candida* spp. colonization and IAC. Positivity of the BDG test (cutoff 80 pg/mL) showed the highest sensitivity (76.7 %) and negative predictive value (94.1 %) as compared to other assays, although CAGTA showed a higher specificity (64.3 %) and a lower sensitivity (53.3 %) than BDG but the specificity of BDG improved when increasing the cutoff from 80 pg/mL to 200 pg/mL. C-PCR had a sensitivity of 84 % and a specificity of 32.9 %. All 20 C-PCR healthy controls were negative. The remaining tests showed a much lower reliability for the diagnosis of IC.

**Table 4:** Number of patients with BDG (cutoff 80, 100 and 200 pg/mL), CAGTA, MANNAN biomarkers and *Candida* PCR positives used alone in the five study groups

	*Candida* spp colonization					
	NCI	LGCC	HGCC	IAC	Candidemia	*P*
	*N* = 48	*N* = 130	*N* = 24	*N* = 20	*N* = 11	
BDG ≥ 80 pg/mL, no. (%)	16/46 (34.8)^a^	50/124 (40.3)^a^	17/24 (70.8)^b^	15/20 (75.0)^b^	8/10 (80.0)^b^	< .001
BDG ≥ 100 pg/mL, no. (%)	16/46 (34.8)^a^	45/125 (36.0)^a^	14/24 (58.3)^a,b^	13/20 (65.0)^b^	8/10 (80.0) ^b^	.004
BDG ≥ 200 pg/mL, no. (%)	10/47 (21.3)^a^	20/128 (15.6)^a^	11/24 (45.8)^b^	10/20 (50.0)^b^	8/10 (80.0)^b^	< .001
CAGTA positive, no. (%)	10/47 (21.3)^a^	44/128 (34.4)^a^	17/24 (70.8)^b^	8/20 (40.0)^a,b^	8/10 (80.0)^b^	< .001
Mannan-Ag positive, no. (%)	10/48 (20.8)^a^	40/127 (31.5)^a^	15/24 (62.5)^b^	8/20 (40.0)^a,b^	5/10 (50.0)^a,b^	.007
Mannan-Ab positive, no. (%)	6/48 (12.5)	12/128 (9.4)	4/24 (16.7)	5/20 (25.0)	3/11 (27.3)	.126
C-PCR positive, no. (%)	14/23 (60.9)	37/54 (68.5)	6/8 (75.0)	12/14 (85.7)	9/11(81.8)	.525

Values are frequencies and percentages. Different superscripts (^a,b^) indicate nominally significant differences (*P* < 0.05). Biomarker positives: two determinations consecutives positives (≥ cutoff). C-PCR positive: one determination positive

*NCI* neither colonized nor infected, *LGCC* low-grade *Candida* spp. colonization, *HGCC* high-grade *Candida* spp. colonization, *IAC* intra-abdominal candidiasis, *BDG* (1-3)-ß-D-glucan, *CAGTA Candida albicans* germ tube antibody, *mannan-Ag* mannan antigen, *mannan-Ab* mannan antibody, *C-PCR* PCR-based *Candida* detection

**Table 5:** Performances of BDG (cutoff 80, 100 and 200 pg/mL), CAGTA, MANNAN biomarkers and C-PCR used alone for IC diagnosis

Invasive candidiasis	Sensitivity %	Specificity %	NPV %	PPV %
	(95 % CI)	(95 % CI)	(95 % CI)	(95 % CI)
BDG ≥ 80 pg/mL	76.7 (57.7–90.1)	57.2 (49.9–64.3)	94.1 (89.1–96.8)	21.7 (17.7–26.4)
BDG ≥ 100 pg/mL	70.0 (50.6–85.3)	61.5 (54.3–68.4)	93.0 (88.4–95.9)	21.9 (17.3–27.3)
BDG ≥ 200 pg/mL	60.0 (40.6–77.3)	79.4 (73.1–84.8)	92.9 (89.4–95.4)	30.5 (22.7–39.6)
CAGTA positive	53.3 (34.3–71.7)	64.3 (57.2–71.0)	90.1 (86.0–93.2)	18.4 (13.3–24.8)
Mannan-Ag positive	43.3 (25.5–62.6)	67.3 (60.3–73.8)	88.7 (85.0–91.6)	16.7 (11.3–24.0)
Mannan-Ab positive	25.8 (11.9–44.6)	89.0 (83.8–93.0)	88.6 (86.2–90.6)	26.7 (15.1– 42.6)
C-PCR positive	84.0 (63.9–95.5)	32.9 (23.1–44.0)	87.5 (73.1–94.8)	26.9 (22.7–31.6)

Values are frequencies and percentages. Biomarker positives: two determinations consecutives positives (≥ cutoff). C-PCR positive: one determination positive

*NCI* neither colonized nor infected, *LGCC* low-grade *Candida* spp. colonization, *HGCC* high-grade *Candida* spp. colonization, *IAC* intra-abdominal candidiasis, *BDG* (1-3)-ß-D-glucan, *CAGTA Candida albicans* germ tube antibody, *mannan-Ag* mannan antigen; *mannan-Ab* mannan antibody, *C-PCR* PCR-based *Candida* detection

### Diagnostic value of combined use of BDG (cutoffs 80, 100 and 200 pg/mL), CAGTA, mannan-Ag, mannan-Ab, and C-PCR

Positivity of the BDG test for the three thresholds (or cutoffs) combined with positive results of the CAGTA, mannan-Ag, and mannan-Ab tests, as well as positivity of the CAGTA test combined with positive results of the mannan-Ag and mannan-Ab tests were significantly more frequent among patients with IAC, candidemia, and high-grade *Candida* spp. colonization than among those with low-grade *Candida* colonization and neither colonized nor infected (Table 6). Similar results were obtained for the combinations of positivities of C-PCR and mannan-Ag as well as mannan-Ag and mannan-Ab, which were also significantly more frequent among patients with IC and high-grade colonization.

**Table 6:** Number of patients with BDG (cutoff 80, 100 and 200 pg/mL) CAGTA, MANNAN biomarkers and *Candida* PCR positives used combined in the five study groups

		*Candida spp. colonization*					
		NCI	LGCC	HGCC	IAC	Candidemia	*P*
		*N* = 48	*N* = 130	*N* = 24	*N* = 20	*N* = 11	
BDG ≥80 pg/mL	CAGTA	21 (43.8)^a^	75 (57.7)^a^	21 (87.5)^b^	18 (90.0)^b^	10 (90.9)^b^	< .001
	Mannan-Ag	21 (43.8)^a^	71 (54.6)^a,c^	21 (87.5)^b^	16 (80.0)^b^	9 (81.8)^b,c^	<.001
	Mannan-Ab	21 (43.8)^a^	61 (46.9)^a^	19 (79.2)^b,c^	15 (75.0)^c^	8 (72.7)^a,c^	.002
	C-PCR	14/23 (60.9)	32/54 (59.3)	5/8 (62.5)	11/14 (78.6)	8/11 (72.7)	.724
BDG ≥100 pg/mL	CAGTA	21 (43.8)^a^	72 (55.4)^a^	20 (83.3)^b^	16 (80.0)^b^	10 (90.9)^b^	< .001
	Mannan-Ag	21 (43.8)^a^	68 (52.3)^a, c^	19 (79.2)^b^	14 (70.0)^a, b, c^	9 (81.8)^c^	.009
	Mannan-Ab	21 (43.8)	57 (43.8)	16 (66.7)	13 (65.0)	8 (72.7)	.053
	C-PCR	14/23 (60.9)	28/54 (51.9)	5/8 (62.5)	10/14 (71.4)	8/11 (72.7)	.606
BDG ≥200 pg/mL	CAGTA	16 (33.3)^a^	54 (41.5)^a^	19 (79.2)^b^	14 (70.0)^b^	10 (90.9)^b^	< .001
	Mannan-Ag	15 (31.2)^a^	52 (40.0)^a^	18 (75.0)^b^	13 (65.0)^b^	9 (81.8)^b^	< .001
	Mannan-Ab	13 (27.1)^a^	33 (25.4)^a^	13 (54.2)^b^	12 (60.0)^b^	8 (72.7)^b^	< .001
	C-PCR	10/23 (43.5)	21/54 (38.9)	3/8 (37.5)	10/14 (71.4)	8/11 (72.7)	.089
CAGTA positive	Mannan-Ag	15 (31.2)^a^	63 (48.5)^b^	21 (87.5)^c^	11 (55.0)^a,b^	10 (90.9)^c^	< .001
	Mannan-Ab	14 (29.2)^a^	53 (40.8)^a,c^	18 (75.0)^b^	12 (60.0)^b,c^	8 (72.7)^b^	< .001
	C-PCR	8/23 (34.8)	25/54 (46.3)	5/8 (62.5)	6/14 (42.9)	8/11 (72.7)	.278
C-PCR positive	Mannan-Ag	5/23 (21.7)^a^	21/54 (38.9)^a^	5/8 (62.5)^a,b^	7/14 (50.0)^a^	8/11 (72.7)^b^	.037
	Mannan-Ab	8/23 (34.8)	19/54 (35.2)	1/8 (12.5)	7/14 (50.0)	8/11 (72.7)	.073
Mannan-Ag positive	Mannan-Ab	13 (27.1)^a^	51 (39.2)^a,b^	17 (70.8)^c^	12 (60.0)^b,c^	5 (45.5)^a,c^	.003

Values are frequencies and percentages. Combined biomarkers and C-PCR: two consecutive determinations positives or/and at least one of each biomarkers (in one determination) positive. C-PCR: one determination positive

*NCI* neither colonized nor infected, *LGCC* low-grade *Candida* spp. colonization, *HGCC* high-grade *Candida* spp. colonization, *IAC* intra-abdominal candidiasis, *BDG* (1-3)-ß-D-glucan, *CAGTA Candida albicans* germ tube antibody, *mannan-Ag* mannan antigen, *mannan-Ab* mannan antibody, *C-PCR* PCR-based *Candida* detection

Different superscripts (a,b,c) indicate nominally significant differences (*P* < .05)

As shown in Table 6, in the group of candidemia, the number of 10 patients with BDG and CAGTA positivities remained unchanged despite increasing the cutoff of BDG from 80 pg/mL to 200 pg/mL. By contrast in the group of IAC, the number of patients with BDG and CAGTA positivities decreased from 18 at the cutoff of 80 pg/mL to 16 at 100 pg/mL and 14 at 200 pg/mL. In the group of high-grade *Candida* spp. colonization, even increasing the cutoff from 80 pg/mL to 200 pg/mL, 19 of the 24 patients continued to show positivities of both BDG and CAGTA biomarkers. When the group of neither colonized nor infected or with low-grade *Candida* spp. colonization was analyzed, the number of patients with positive BDG and positive CAGTA decreased from 96 (53.9 %) for a cutoff of 80 pg/mL to 70 (39.3 %) for a cutoff of 200 pg/mL.

The combination of BDG positivity with positive values of other markers showed the highest sensitivities (Table 7). BDG (cutoff 80 pg/mL) plus CAGTA showed a sensitivity of 90.3 % and a negative predictive value of 96.6 %, although the specificity was only 42.1 %, increasing to 55.9 % when the cutoff of BDG was 200 pg/mL. The combination of BDG and mannan-Ag showed high sensitivities and negative predictive values (80.6 % and 93.7 %, respectively), but a low specificity (44.1 %). C-PCR showed the best specificities when combined with mannan-Ag and mannan-Ab (63.5 % and 78.7 %, respectively). The remaining combinations were less relevant.

**Table 7:** Performances of BDG (cutoff 80, 100 and 200 pg/mL), CAGTA, MANNAN biomarkers and C-PCR used combined for IC diagnosis

Invasive candidiasis		Sensitivity %	Specificity %	NPV %	PPV %
		(95 % CI)	(95 % CI)	(95 % CI)	(95 % CI)
BDG ≥80 pg/mL	CAGTA	90.3 (74.2–98.0)	42.1 (35.2–49.2)	96.6 (90.5–98.8)	19.3 (16.9–22.0)
	Mannan-Ag	80.6 (62.5–92.5)	44.1 (37.1–51.2)	93.7 (87.7–96.9)	18.1 (15.2–21.5)
	Mannan-Ab	74.2 (42.9–57.1)	50.0 (42.9–57.1)	92.7 (87.2–95.9)	18.5 (15.1–22.6)
	C-PCR	76.0 (54.9–90.6)	40.0 (29.5–51.2)	85.0 (72.9–92.3)	27.1 (22.0–33.0)
BDG ≥100 pg/mL	CAGTA	83.9 (66.3–94.5)	44.1 (37.1–51.2)	94.7 (88.7–97.6)	18.7 (15.9–21.9)
	Mannan-Ag	74.2 (55.4–88.1)	46.5 (39.5–53.7)	92.2 (86.4–95.6)	17.6 (14.3–21.4)
	Mannan-Ab	67.7 (48.6–83.3)	53.5 (46.3–60.5)	91.5 (86.5–94.8)	18.3 (14.4–22.9)
	C-PCR	72.0 (50.6–87.9)	44.7 (33.9–55.9)	84.4 (73.5–91.4)	27.7 (21.9–34.3)
BDG ≥200 pg/mL	CAGTA	77.4 (58.9–96.9)	55.9 (48.8–62.9)	94.2 (89.3–96.9)	21.2 (17.4–25.6)
	Mannan-Ag	71.0 (52.0–85.8)	57.9 (50.8–64.8)	92.9 (88.1–95.8)	20.6 (16.4–25.5)
	Mannan-Ab	64.5 (45.4–80.8)	70.8 (64.0–77.0)	92.9 (88.9–95.5)	25.3 (19.5–32.2)
	C-PCR	72.0 (50.6–87.9)	60.0 (48.8–70.5)	87.9 (79.1–93.3)	34.6 (27.0–43.1)
CAGTA positive	Mannan-Ag	67.7 (48.6–83.3)	51.0 (43.9–58.1)	91.2 (85.9–94.6)	17.5 (13.8–21.9)
	Mannan-Ab	64.5 (45.4–80.8)	57.9 (50.8–64.8)	91.4 (86.7–94.5)	19.0 (14.8–24.2)
	C-PCR	56.0 (34.9–75.6)	55.3 (44.1–66.1)	81.0 (72.5–87.4)	26.9 (19.5–35.9)
C-PCR positive	Mannan-Ag	60.0 (38.7–78.9)	63.5 (52.4–73.7)	84.4 (76.5–90.0)	32.6 (24.0–42.5)
	Mannan-Ab	54.8 (36.0–72.7)	78.7 (72.4–84.1)	91.9 (88.4–94.4)	28.3 (20.7–37.5)
Mannan-Ag positive	Mannan-Ab	54.8 (36.0–72.7)	59.9 (52.8–66.7)	89.6 (85.2–92.8)	17.3 (12.8–23.1)

Combined biomarkers and C-PCR: two consecutive determinations positive or/and at least one of the biomarkers (in one determination) positive. C-PCR: one determination positive

*BDG* (1-3)-ß-D-glucan, *CAGTA Candida albicans* germ tube antibody, *mannan-Ag* mannan antigen, *mannan-Ab* mannan antibody, *C-PCR* PCR-based *Candida* detection

In the association of positive BDG and positive CAGTA, which was the combination with the highest diagnostic performance, the sensitivity decreased and the specific increased as the cutoffs changed from 80 pg/mL to 100 pg/mL and to 200 pg/mL.

## Discussion

Because of difficulties in the diagnosis IC in ICU settings, up to two thirds of critically ill patients with suspicion of fungal infection are given empirical antifungals [17, 18], a strategy with a well-recognized negative impact on adverse effects, risk of resistances [5], length of stay, patient’s outcome, and health care costs [19, 20]. In the present series, 51 % of patients received SAT and 75.6 % of them did not have documented IC. Therefore, the availability of accurate tests to support either the initiation or discontinuation of SAT is a decision of crucial importance. The clinician can prescribe SAT if two consecutive [21–26] or more positive BDG determinations within 48 hours are present, or may stop SAT when BDG and CAGTA are negative in a single sample due to the high negative predictive value of this combination. Also, performance of BDG as a complementary test to blood cultures is currently under the scope of various clinical recommendations [27–29]. Bailly et al. [30] have recently demonstrated that in the absence of proven IC empiric SAT treatment could be safely stopped after 5 days of therapy without apparent deleterious effect on day-28 mortality.

According to the present findings, positive BDG and positive CAGTA levels in a single sample or at least positivity of one of the two biomarkers in two consecutive samples was the best way of characterizing IC diagnosis in non-neutropenic ICU patients with SAC. By contrast, individual or combined positivities of C-PCR, mannan-Ag, and mannan-Ab were of little value for discriminating IC from *Candida* spp. colonization. These results are relevant in daily practice for making decisions in complex clinical care settings.

Different studies have assessed the clinical value of CAGTA. The group of Martinez-Jiménez et al. [31] showed that the presence of a positive CAGTA test in a sample from a patient with candidemia suggests deep-seated candidiasis, and more recently, provided evidence that the combination of CAGTA and BDG, or CAGTA and mannan-Ag, had high negative predictive values [32]. In 63 ICU patients and 37 non-ICU patients treated with antifungals, these authors achieved a sensitivity of 96.7 % and negative predictive value of 97.1 % when the combination (BDG and CAGTA at 1/60 cutoff) was positive on days 0, +3, and +5 after starting antifungal therapy. In this study, serial determination of BDG and CAGTA could be used to stop safely antifungals in 31 % of patients receiving empirical antifungal therapy both in ICU and non-ICU wards [33].

The advantage of combining BDG and CAGTA for accurate diagnosis of IC is consistent with our findings in selected ICU patients with SAC, with a sensitivity of 90.3 % and a negative predictive value of 96.6 %. Although the specificity was only 42.1 %, the combination of BDG and CAGTA was positive in 21 of 24 patients with high-grade *Candida* spp. colonization, which probably indicates a hidden IC or a high probability of developing candidemia, two circumstances in which starting empirical antifungals would be justified. Also, the high negative predictive value could be useful for excluding *Candida* spp. infection in patients receiving empirical SAT.

The combination of mannan-Ag and mannan-Ab has been also recommended [28, 34] but results of clinical studies are not very encouraging, with a diagnostic specificity of 51 % and sensitivity of 77 % in a retrospective analysis of 162 patients of whom 91 had proven IC [35]. In samples from 31 patients with candidemia and 50 patients with bacteremia, the use of mannan-Ag and mannan-Ab alone showed sensitivities of 64.3 % and 61.5 %, specificities of 95.7 % and 65.8 %, and negative predictive values of 81.8 % and 71.4 %, respectively [32]. In our study, positive mannan-Ag tests were more frequent in patients with high-grade *Candida* colonization (65.5 %) and candidemia (50 %), whereas the rate of positive mannan-Ab tests was low in all study groups. However, both tests had a low sensitivity for diagnosing IC possibly in relation to the limited capacity to produce specific antibodies in the presence of immunosuppression [9, 33].

Currently, molecular techniques are increasingly used to diagnose invasive fungal infections [8, 9]. Results of a meta-analysis with 54 studies and 4694 patients, PCR positivity rates among patients with proven or probable IC were 85 %, while blood cultures were positive for 38 % [36]. In 63 ICU patients with suspected IC (eventually confirmed in 27) and 40 healthy controls, the sensitivity, specificity, positive predictive value and negative predictive value of MRT-PCR for the diagnosis of IC were 96.3 %, 97.3 %, 92.8 % and 98.7 %, respectively. Also, in deep-seated IC, the sensitivity of PCR was 90.9 % vs*.* 45.4 % for blood cultures (*P* = 0.06) [13]. In our study, positive PCR tests were more frequent in patients with IC and high-grade *Candida* colonization, although the diagnostic accuracy was low. However, wide standardization of molecular tests has not been reached and results should be considered with caution.

Other studies have examined the combinations of biomarkers with inconsistent results. Nguyen et al. [37] in blood samples collected prospectively from 55 patients with IC and 73 controls, PCR and BDG were similar for diagnosing candidemia (59 % vs*.* 68 %) but PCR was more sensitive for deep-seated candidiasis (89 % vs. 53 %; *P* = 0.004), and both (PCR and BDG) were more sensitive than blood cultures among patients with deep-seated candidiasis. Held et al. [38] evaluated the usefulness of BDG, mannan-Ag with/without mannan-Ab, and Cand-Tec Candida antigen in a case-control study. The combination of BDG and mannan-Ag was superior to the other combinations with 89.3 % sensitivity and 85.0 % specificity. In a previous study of our group in ICU patients with SAC [20], BDG levels > 259 pg/mL combined with CAGTA positive results accurately discriminated *Candida* spp. colonization from IC (sensitivity 90.3 %, specificity 54.8 %, and negative predictive value 93.9 %).

This study has also its limitations. First, the number of IC was small and this is important because we think that BDG and CAGTA behave differently in candidemia and in deep-seated candidiasis, such as IAC. The number of patients was not sufficient for a reliable analysis of the differences in biomarkers accuracy between IAC and candidemia. Second, although we applied strict criteria for the diagnose IAC, *Candida* spp. isolation in peritoneal exudates is difficult and the role of unregistered polymicrobial cultures is hard to establish. Third, the BDG and PCR tests were performed in frozen samples, so that false negative results are possible due to instability of the samples. Fourth, the presence of intestinal mucositis may facilitate the translocation of *Candida* spp. through the gastrointestinal barrier and eventually may interfere with BDG measurements. Finally, we should take into account the potential impact of echinocandins on BDG synthesis because, in the current study, 119 patients, approximately half of the studied patients, received SAT and nearly 60 % of them were treated with echinocandins.

Our study demonstrates that BDG, in contrast to other methods, is the best biomarker to be used in the diagnosis of IC in the critically ill patient with SAC. When BDG is used in combination with CAGTA, its diagnostic performance increases notably. The stratification of colonized patients (in high-grade and low-grade) and of IC (in candidemia and IAC) allowed us to observe that behavior of these biomarkers is different in these clinical scenarios, particularly when BDG with different cutoffs is used. The association of BDG and CAGTA positivities is an excellent diagnostic tool for candidemia, independently of the cutoff value of BDG, but this was not the case for IAC where the number of patients with BDG and CAGTA positivities decreases as the cutoff value of BDG increases. In cases of high-grade *Candida* spp. colonization, the percentage of patients with both BDG and CAGTA positive results decreases slightly (two patients) when the BDG cutoff increases, which can raise the possibility of a very likely presence of “occult” IC (especially candidemia). From a practical clinical point of view, it may be stated that in critically ill patients with SAC and BDG/CAGTA positivity, with a cutoff of BDG of ≥ 80 pg/mL, the need to start antifungal treatment should be considered, covering the three aforementioned possible clinical scenarios. Another practical issue of combined BDG and CAGTA positivity is the high negative predictive value (96.6 %), which would raise the possibility of stopping an empirical antifungal therapy. However, in patients without *Candida* colonization or with low-grade *Candida* spp. colonization, the association of BDG and CAGTA is positive in 53.9 % of patients, which logically decreases to 39.3 % when a BDG cutoff of 200 pg/mL is used.

## Conclusions

In selected, non-neutropenic critically ill patients with SAC, the combination of BDG and CAGTA positivities in a single determination or at least one of the two biomarkers positive in two consecutive samples, allowed discriminating between IC and the groups of low-grade and high-grade *Candida* colonization as well as neither colonized nor infected. Other tests including PCR *Candida* DNA detection and serum levels of mannan-Ag and mannan-Ab alone or combined did not improve the diagnostic yield. These clinically relevant findings can be exploited to tailor empirical systemic antifungal therapy in ICU patients with SAC.

## Abbreviations

Ab: antibody
AEMPS: Spanish Agency for Medicines and Health Care Products
Ag: antigen
APACHE II: Acute Physiology and Chronic Health Evaluation
BDG: 1 → 3)-ß-D-glucan
CAGTA: *Candida albicans* germ tube antibody
C-PCR: Candida polymerase chain reaction
FEV: forced expiratory volume
FVC: forced vital capacity
HIV: human immunodeficiency virus
IAC: intra-abdominal candidiasis
IC: invasive candidiasis
ICU: intensive care unit
MRT-PCR: multiplex quantitative real-time polymerase chain reaction
PCR: polymerase chain reaction
SAC: severe abdominal conditions
SAT: systemic antifungal therapy
SOFA: Sepsis-related Organ Failure Assessment
