# Erratum to: Contribution of Candida biomarkers and DNA detection for the diagnosis of invasive candidiasis in ICU patients with severe abdominal conditions

**DOI:** 10.1186/s13054-017-1686-1

**Published:** 2017-05-15

**Authors:** Cristóbal León, Sergio Ruiz-Santana, Pedro Saavedra, Carmen Castro, Ana Loza, Ismail Zakariya, Alejandro Úbeda, Manuel Parra, Desirée Macías, José Ignacio Tomás, Antonio Rezusta, Alejandro Rodríguez, Frederic Gómez, Estrella Martín-Mazuelos, The Cava Trem Study Group

**Affiliations:** 1 Intensive Care Unit, Hospital Universitario de Valme, Universidad de Sevilla, Avenida Bellavista s/n, 41014 Sevilla, Spain; 2 Intensive Care Unit, Hospital León et al. Critical Care (2016) 20:149 Page 13 of 14 Universitario Dr. Negrín, Universidad de Las Palmas de Gran Canaria, Las Palmas de Gran Canaria, Spain; 3 Mathematics Department, Universidad de las Palmas de Gran Canaria, Las Palmas de Gran Canaria, Spain; 4 Clinical Unit of Microbiology and Infectious Diseases, Hospital Universitario de Valme, Universidad de Sevilla, Sevilla, Spain; 5 Intensive Care Unit, Hospital Punta de Europa, Algeciras, Cádiz, Spain; 6 Intensive Care Unit, Hospital Universitario Miguel Servet, Zaragoza, Spain; 7 Service of Microbiology, Hospital Universitario Miguel Servet, Zaragoza, Spain; 8 Critical Care Department, Hospital Universitari Joan XXIII, Tarragona, Spain; 9 Service of Microbiology, Hospital Universitari Joan XXIII, Tarragona, Spain

## Erratum

Unfortunately this article [1] was published with an error. The following information was omitted: This study has received financial support from the national I + D + i 2013-2016 plan and been co-financed by the ISCII-General Subdirection of Evaluation and Promotion of Research and by the European Funds of Regional Development (FEDER) under Project reference PI13/01168.
